# Use of bioreactor systems in the propagation of forest trees

**DOI:** 10.1002/elsc.201900041

**Published:** 2019-09-30

**Authors:** Nieves Vidal, Conchi Sánchez

**Affiliations:** ^1^ Instituto de Investigaciones Agrobiológicas de Galicia CSIC Santiago de Compostela Spain

**Keywords:** axillary shoots, continuous immersion, rooting, somatic embryos, temporary immersion

## Abstract

Plant biotechnology can be used to conserve the germplasm of natural forests, and to increase the productivity and sustainability of plantations. Both goals imply working with mature trees, which are often recalcitrant to micropropagation. Conventional in vitro culture uses closed containers and gelled medium with sugar supplementation. Bioreactor culture uses liquid medium and usually incorporates aeration. The increased absorption of nutrients via the liquid medium together with the renewal of the air inside the bioreactors may improve the physiological state of the explants. In this review, we will explore the feasibility of using bioreactors to overcome the recalcitrance of many trees to micropropagation and/or to decrease the cost of large‐scale propagation. We will focus on the recent use of bioreactors during the multiplication, rooting (plant conversion in the case of somatic embryos), and acclimation stages of the micropropagation of axillary shoots and somatic embryos of forest trees (including some shrubs of commercial interest), in both temporary and continuous immersion systems. We will discuss the advantages and the main obstacles limiting the widespread implementation of bioreactor systems in woody plant culture, considering published scientific reports and contributions from the business sector.

**Abbreviations**
CIS: continuous immersion system
PAM: photoautotrophic micropropagation
RITA: recipient for automated temporary immersion
SS: semisolid medium
TIS: temporary immersion system

## 1 INTRODUCTION

From an ecological point of view, forest species are essential for the preservation of ecosystems and the general equilibrium of the biosphere. From a human perspective, forest species also represent a source of raw materials in economically important sectors such as the buildings and paper industries. Trees provide food, resins, and medicinal products, and they can also be used in phytoremediation. Despite the difficulties associated with the economic quantification of this contribution, forest trees play a central role in maintaining landscapes and in establishing recreation areas.

Because of the wide range of possible uses for trees, many areas formerly occupied by natural forests have been transformed into plantations managed for productive purposes. In this context, scientific approaches should consider trees in natural forests and also the “domesticated” trees used in plantations 1. Increasing the productivity of plantations should decrease the pressure to allocate more land for this purpose, thus mitigating the associated risks, such as replacement of native species and potential long‐term loss of diversity at the landscape level 2, 3.

Natural forests are extraordinarily valuable as reservoirs of genetic diversity. In addition, underused “wild” woody plants will probably become more important in the fight against climate change or other environmental problems in the near future, and they may also be discovered to be important in new profitable applications. For these wild trees, germplasm conservation should be the main scientific focus 4, whereas for trees used in plantations the priority goals should be to balance the increased productivity with environmental sustainability.

Plant biotechnology (including in vitro culture) can be used to preserve valuable genotypes, to propagate superior material on a large scale, to develop physiological studies, and even to obtain genetically transformed trees. Protocols have been developed for the micropropagation of many different tree species 5, 6, 7, but recalcitrance to in vitro culture and some characteristics of forest trees hinder the applicability of these protocols to large‐scale propagation and to the preservation of elite trees.

The rapid improvement of trees through sexual breeding is restricted by the high heterozygosity and the long life cycles of forest trees 8. However, vegetative propagation enables the capture of additive and non‐additive genetic gain derived by selection 3, 9. Trees do not exhibit stable desirable traits until they have reached maturity. In order to maximize genetic gain, phenotypically characterized adult trees should be selected and used for micropropagation 10, 11, 12. However, vegetative propagation of woody plants becomes increasingly difficult as the trees age 13, 14. Despite major advances in forest biotechnology, clonal regeneration by somatic embryogenesis or organogenesis remains difficult for many tree species and is often limited to juvenile explants 10. In comparison with juvenile material, mature plants usually prove more recalcitrant to the establishment of aseptic and reactive cultures, multiplication (usually hindered by the several months required for culture stabilization), adventitious rooting (or plant conversion in the case of somatic embryos), and acclimation 15, 16, 17, 18. Moreover, genotypic differences within tree species in relation to the response to each stage of micropropagation suggest that current protocols are not efficient enough for commercial application, which requires homogenous performance for a wide spectrum of proven genotypes 19. Although it appears from the literature that micropropagation protocols have been successfully established for various forest tree species, this is probably only true at an experimental scale and not operationally, as further development of micropropagation remains hindered by serious limitations, as highlighted by Monteuuis 20.

In this review, we will explore the feasibility of using bioreactors to overcome these limitations. It has been claimed that the increased absorption of nutrients via the liquid medium, together with the renewal of the air inside the bioreactors may improve the physiological state of the explants and make them more competent to undergo rooting and acclimation 21, 22, 23, 24, 25, 26, 27. We will focus on the recent use of bioreactors in the micropropagation of axillary shoots or somatic embryos of forest trees (including some shrubs of commercial interest) during the following stages: i) multiplication, ii) rooting or plant conversion, and iii) acclimation. The advantages and the main obstacles limiting the widespread use of bioreactors in woody plant culture will be discussed, and published scientific reports and contributions from the business sector will be considered. Regarding the type of bioreactor, we will consider both temporary and continuous immersion systems (CIS). In order to stay within the length restrictions for this review paper, we will mainly focus on tree species that form an important part of natural forests or plantations, or that are currently being used for reforestation and afforestation activities. Regarding these trees, we will select those reports in which protocols are sufficiently well developed to be applied to plant production.

**PRACTICAL APPLICATION**

This review has been written as a contribution for the Special Issue Plant Cells and Algae in bioreactors. The aims of this work are: (1) To highlight the specific difficulties for the micropropagation of forest trees, (2) to review the current state of the application of bioreactors to these trees, and (3) to evaluate if using bioreactors is possible to overcome the recalcitrance of some trees for micropropagation.

## 2 BIOREACTORS SYSTEMS FOR THE PROPAGATION OF TREES

The term bioreactor describes large‐scale vessels used for plant biomass production. Bioreactors were first developed for culturing microorganisms, then for plant cell suspensions for secondary metabolite production, and later for plant propagation purposes. The aim of bioreactor application is to provide optimum growth conditions by regulating chemical or physical parameters, in order to achieve either both maximum yield and high quality of the explants, or to keep the production costs as low as possible by integration of automated facilities and simple low‐cost devices 28. Among the many categories in which bioreactors can be classified, here we will distinguish between continuous immersion and temporary immersion bioreactors.

The culture of a plant in a CIS means that the liquid medium is continuously in contact with at least one section of the explant. Stationary immersion of the whole explant usually causes hyperhydricity and malformations, since oxygen concentration in liquid media is often insufficient to meet the respiratory requirements of the submerged tissues 29, 30, 31. Oxygen depletion in plant cells induces oxidative stress, with production of reactive oxygen species, and therefore causes injury to the plant tissue 30. To avoid these problems, oxygen can be provided by agitation and/or aeration, or by maintaining part of the explant in contact with air 31.

Temporary immersion systems (TIS) represent another approach. TIS enable temporary contact between the plants and the liquid medium, thus avoiding continuous immersion and providing adequate oxygen transfer to the cultures 21, 22, 23. The thorough description and functioning of CIS and TIS, as well as the various particular designs are outside the scope of this review. Brief information about the bioreactors most frequently used for the propagation of trees is given below. For detailed information on these topics, readers are referred to several comprehensive reviews 21, 24, 25, 26, 27, 32, 33, 34, 35.

### 2.1 Bioreactors based on continuous immersion

**Figure 1:**
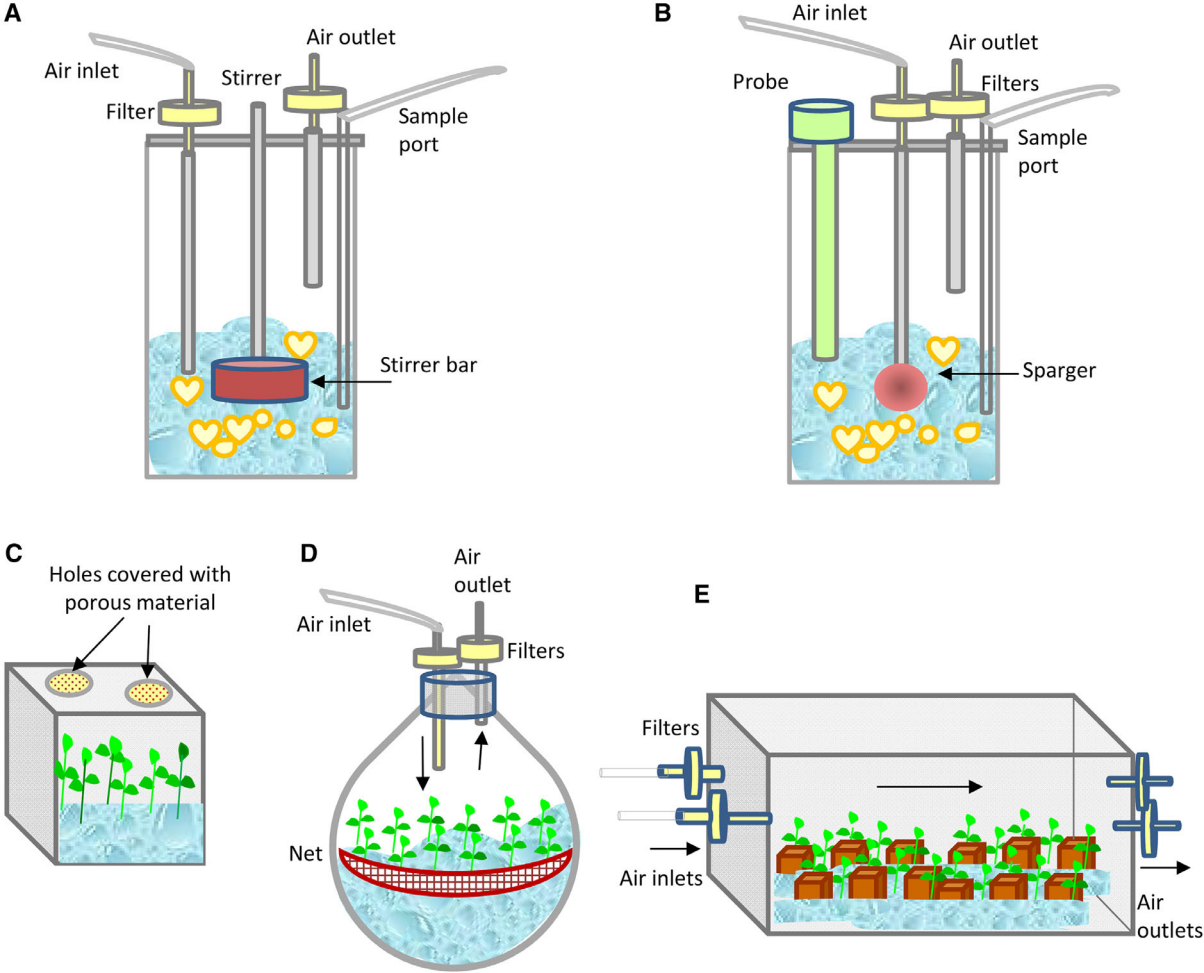
Schemes showing the basic design of some CIS bioreactors used for propagation of trees. (A) Stirred tank, (B) airlift bioreactor, (C) small flask with natural ventilation, (D) balloon with forced ventilation and a net to hold the explants, and (E) large vessel with forced ventilation and porous support material for inserting the explants

Schemes showing the basic design and operation mode of some of the CIS bioreactors used for propagation of trees are shown in Figure 1. The first two bioreactors (Figure 1A and B) correspond to stirred tank and airlift, respectively, which are frequently used to culture somatic embryos. A stirred tank is a mechanically operated bioreactor that consists of an impeller or agitator along with different ports for aeration, medium addition or removal, in order to facilitate liquid circulation, mixing, and distribution of O_2_, and nutrients 26. The airlift is a pneumatic bioreactor equipped with a sparger for forming small bubbles of filtered air that rise through the column of liquid medium thereby aerating and mixing the culture. The key parameters for the efficient use of these bioreactors include control of shear, ease of gas and medium exchange, and maintenance of sterility. Although shear stress is caused in both mechanically and pneumatically operated bioreactors due to mechanical agitation and aeration respectively, its effects are less harmful in airlift vessels 26. Figure 1C and D represents some bioreactors used to culture axillary shoots, as a small vessel with natural ventilation (Figure 1C), a balloon bioreactor with net, in which the explants are partially submerged (Figure 1D), and a large vessel with forced ventilation and porous support material for inserting the explants 36 (Figure 1E). In the first case, air enters the vessel by simple diffusion through membrane filters. This approach can give acceptable results with small containers, but usually it is not suitable for large vessels due to the occurrence of hyperhydricity. The gaseous environment of large vessels (as those represented in Figure 1D and E) can be improved by forced ventilation, i.e. mechanically moving filtered air from the outside to the inside of a culture vessel and vice versa with the aid of an air pump 37. The use of continuous immersion with forced ventilation enables the size of bioreactors to be increased by adapting vessels intended for other uses (i.e. food containers) without the need to implement more complicated TISs, thereby lowering production costs.

### 2.2 Bioreactors based in temporary immersion

**Figure 2:**
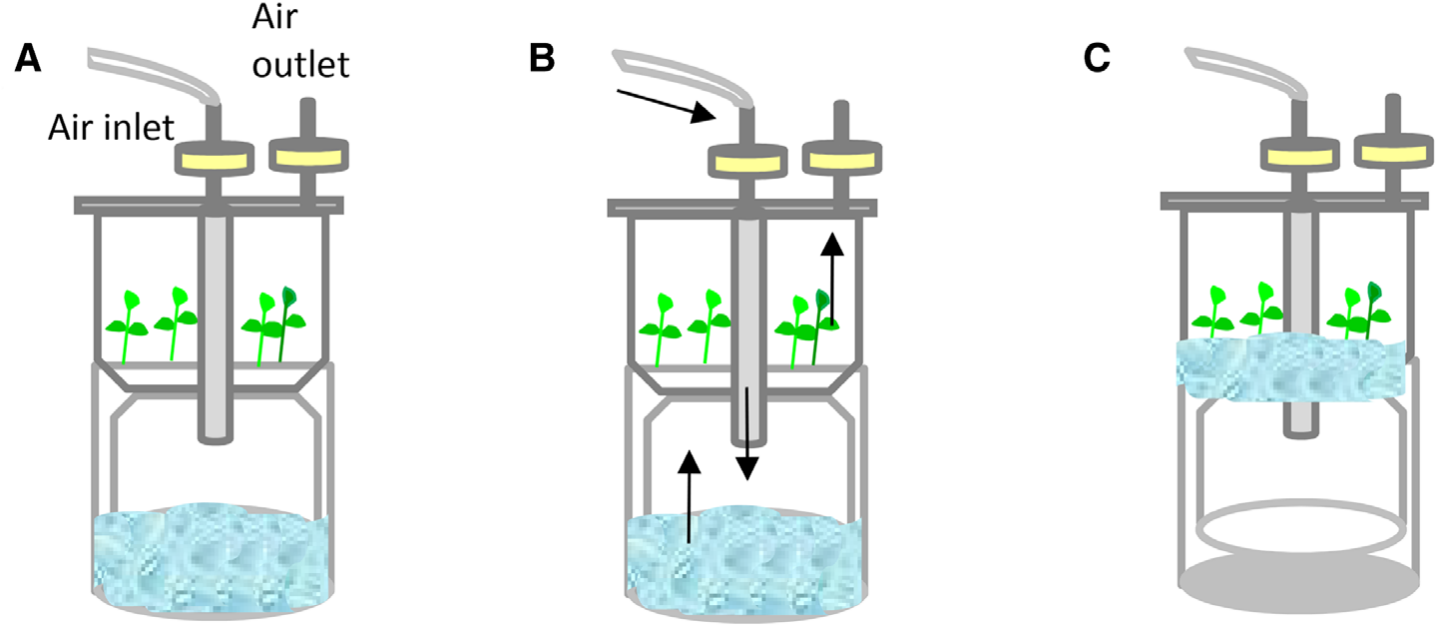
RITA^®^ bioreactor. (A) The pump is off and the liquid medium is in the lower compartment, (B) the pump impulses air through the inlet filter, (C) the overpressure moves the medium up and cause immersion of the explants, as well as air expulsion through the outlet filter. When the pump is off, the medium goes down by gravity

**Figure 3:**
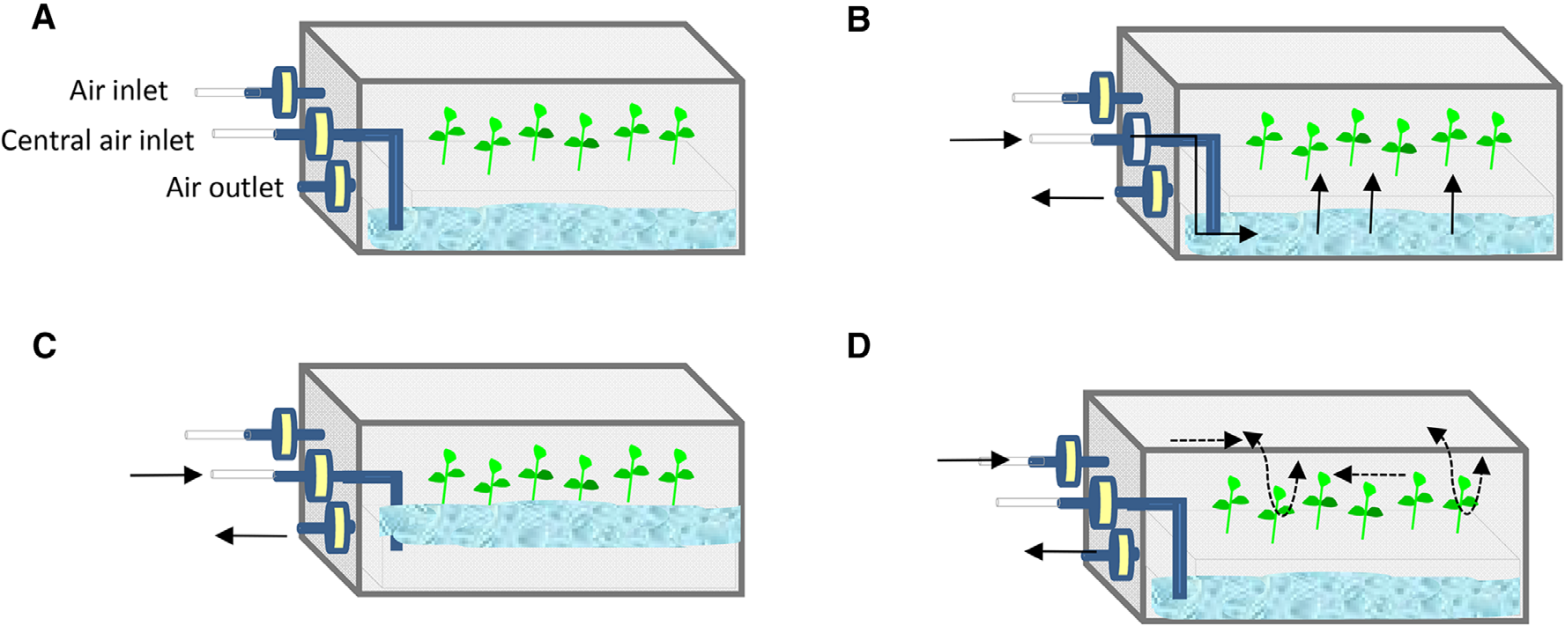
Plantform™ bioreactor. (A) The pump is off and the liquid medium is in the lower compartment, separate from the explants, (B) the pump impulses air through the central inlet filter, (C) the overpressure moves the medium up and cause immersion of the explants, as well as air expulsion through the outlet filter. When the pump is off, the medium goes down by gravity, and (D) additional aerations: the pump impulses air through any of the lateral inlet filters. The air circulates through the chamber containing the explants, but does not cause translocation of the medium

**Figure 4:**
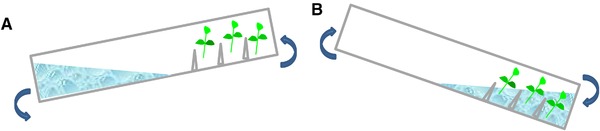
Rocker bioreactor. (A) Due to the angle of the container, the medium is a separate section from the explants and (B) the container moves and with the change of angle the explants are immersed in the medium

**Figure 5:**
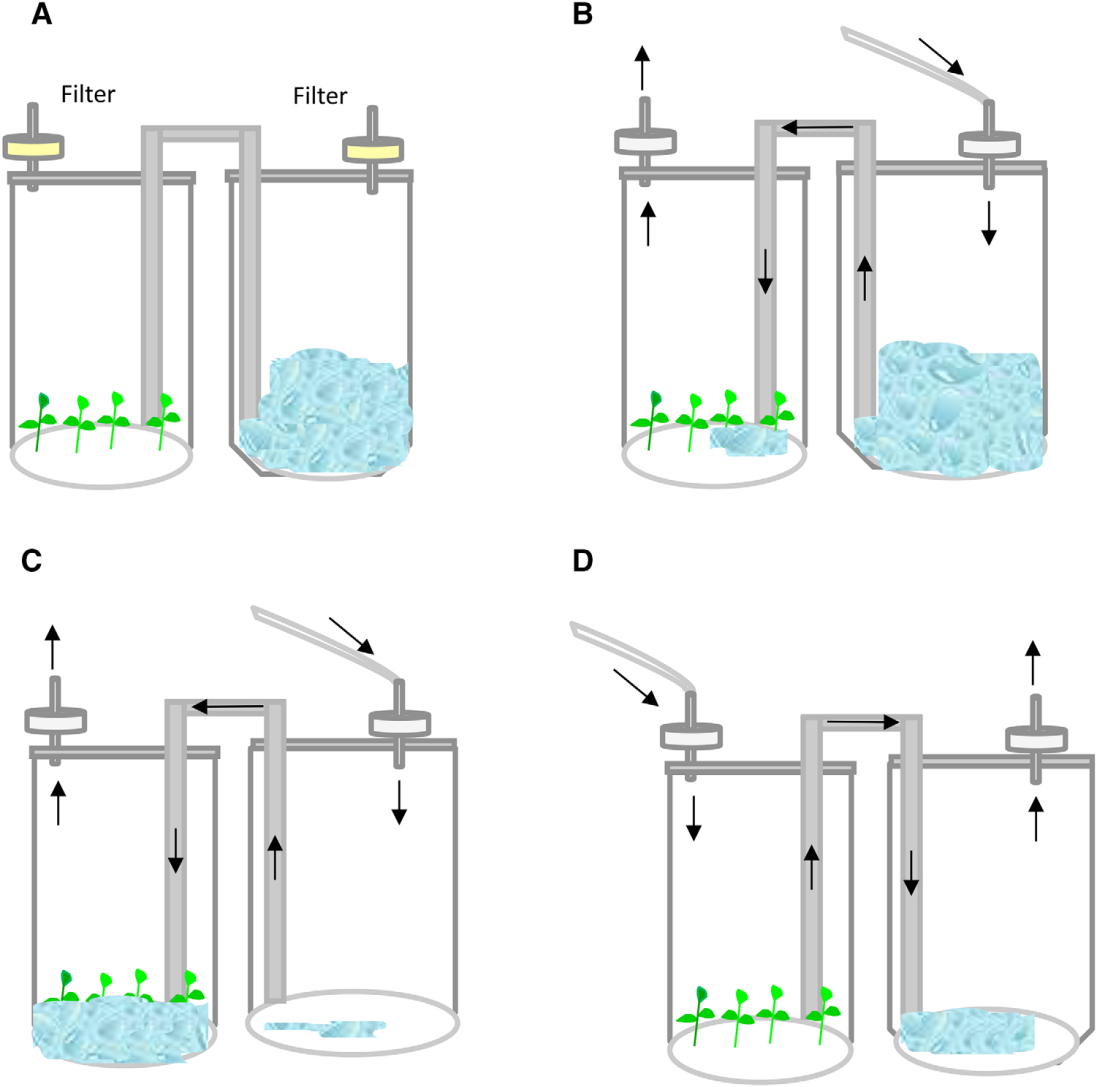
Two‐flask bioreactor. (A) The liquid medium is in a separate flask from the culture vessel that holds the explants, (B) the pump impulses air through the flask containing the medium, forcing its movement to the culture vessel, (C) the medium cause immersion of the explants, as well as air expulsion through the outlet filter, and (D) the pump impulses air through the culture vessel, forcing its movement to the empty flask

**Figure 6:**
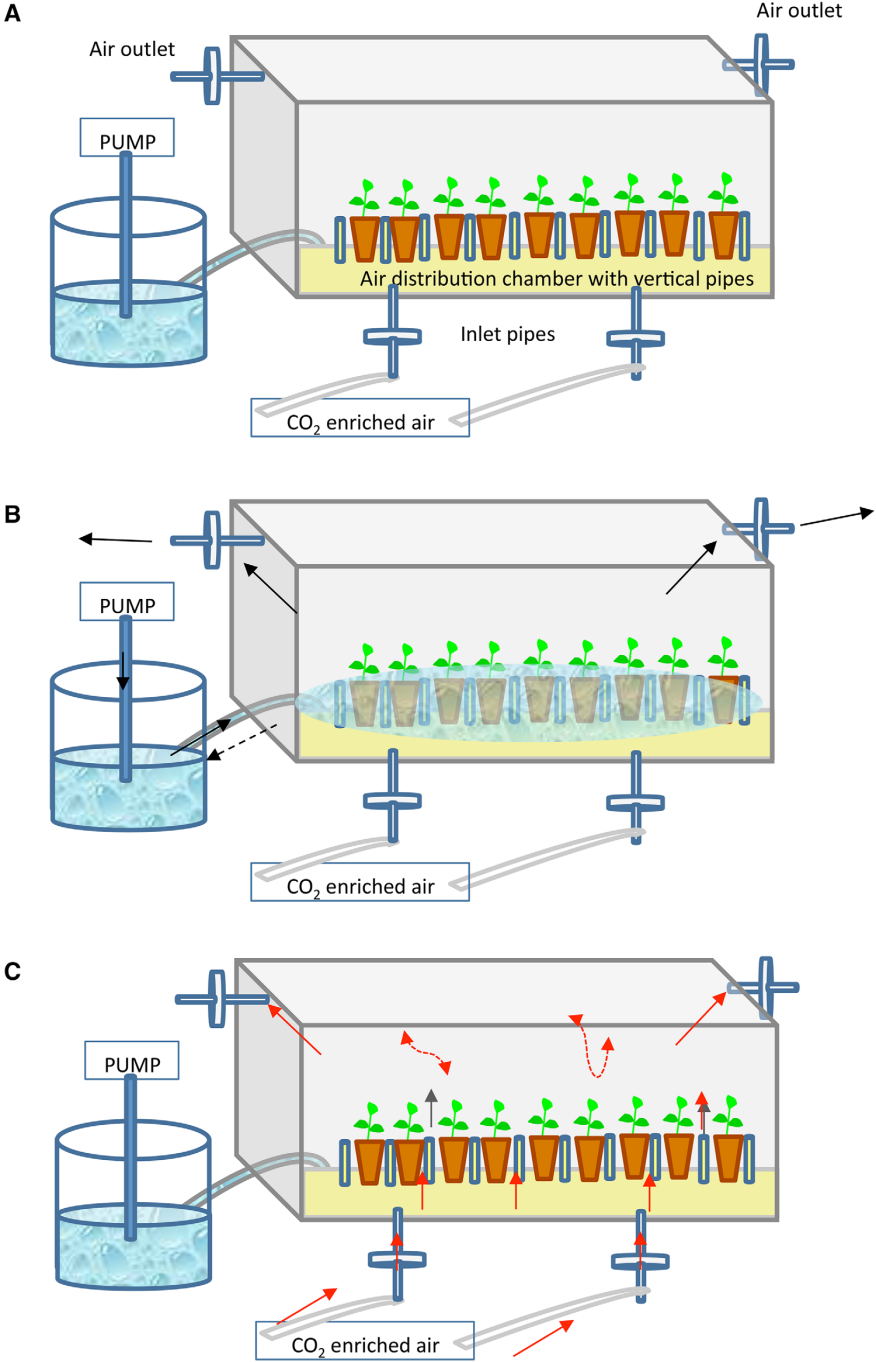
Two‐flask bioreactor with additional forced ventilation, TRI‐bioreactor. (A) The liquid medium is in a reservoir connected with the culture vessel, (B) the pump is switched on to impulse air through the flask containing the medium, forcing its movement into the culture vessel. Once the immersion is completed, the pump is switched off and the medium flows back in the reservoir under gravity, and (C) CO_2_‐enriched air is pumped through the inlet pipes and directed to the culture vessel headspace, without causing translocation of the medium

TIS was first described by Steward in 1952 38, but its massive use began years later, after the studies of Alvard and Teisson 22, 23. The bioreactors most frequently used for micropropagation of trees are mainly those derived from the two‐flask system 39, commercial recipient for automated temporary immersion (RITA)^®^ 23 and plantform™ 40 bioreactors, as well as others like the rocker system 41, 42. Figures 2, 3, 4, 5, 6 show some schemes of these apparatus. In all cases, the explants are placed separately from the liquid medium, either in a different compartment or zone of the same flask (RITA^®^, plantform™, and rocker bioreactors; Figures 2, 3, 4), or in an independent container connected by tubes (two‐flask system, Figures 5 and 6). The explants can be placed either directly on the bioreactor inner surface or by using different support materials (nets, glass beads, rockwool cubes, polyurethane foam, etc.). The medium reaches the explants by mechanical movement of the entire vessel (rocker bioreactors, Figure 4) or by the driven force of filtered air pumped at programmed intervals, as happens in RITA^®^ (Figure 2), plantform™ (Figure 3), and two‐flask bioreactors (Figures 5 and 6). In the latter three cases, pumped air not only enables the contact of the explants with the medium but also causes the renewal of the gaseous atmosphere inside the vessels, thus promoting photoautotrophic behavior 43. Besides variations as regards type of material, size and shape, other features differentiate these designs and may influence their suitability for the micropropagation of specific species. For example, the rigid inner tubes of RITA^®^ apparatus facilitate handling during medium exchange, which may be useful for plants that need several transfers during their culture cycle. Plantform™ vessels are not so easy to manage in these terms, but its arrangement of inlet/outlet holes allows to apply additional aerations independently of those directed to force the movement of the medium. As these additional aerations do not cause immersion of the explants, this system may be useful for plants that are especially prone to hyperhydricity, one of the most frequent hindrances associated to liquid medium 44, 45. Immersion time (duration and frequency) is the most decisive parameter for system efficiency, and once protocols have been optimized, plants cultured by TIS generally show increased vigor and better quality than those grown completely submerged in liquid medium or conventionally in semisolid medium (SS) 21.

## 3 APPLICATION OF BIOREACTORS TO THE MICROPROPAGATION OF TREES

This section summarizes the state of the art on the application of CIS/TIS to the propagation of trees cultured both by axillary shoot propagation and by somatic embryogenesis.

### 3.1 Axillary shoots cultured by continuous immersion

Table 1 summarizes nine studies conducted with shoots immersed continuously in liquid medium. Only five genus are represented, as the use of this system is not extended in the case of trees. Shoots were proliferated by CIS in 78% of references but rooting and acclimation were reported only in 56% of cases. Within the examples cited in Table 1, eucalyptus is the best represented tree (one third of the references). Species of the genus *Eucalyptus*, native to Australia, are amongst the most widely trees grown in forest plantations, despite the widespread public concern originated by the ecological problems associated with its massive use 46, 47. Although eucalyptus are fast growing trees, many of the natural species and hybrids used commercially are recalcitrant to vegetative propagation (carried out either by cuttings or by conventional micropropagation). This recalcitrance, mainly due to poor adventitious rooting, causes in turn high plant losses during acclimation. CIS with natural or forced ventilation have been tested for solving these problems. Shoots of *E. camaldulensis* with their bases continuously exposed to liquid medium were cultured in glass flasks with forced ventilation 48 or in small flasks with natural ventilation 49. In both cases, the multiplication coefficients were higher than obtained in semi‐solid medium, although hyperhydricity was detected 48. This disorder also affected the propagation of apple shoots in a 5 L balloon with a net (Figure 1D), indicating that other parameters beside forced ventilation influence shoot quality 30. Other eucalyptus (*Urophylla* x *Grandis*) were cultured in different vessels as Miracle Pack and Vitron. These vessels were used with natural ventilation in a chamber with CO_2_‐enriched air, in order to achieve a photoautotrophic behavior 50, and rooted and acclimated plantlets were obtained.

**Table 1:** Application of CIS to the propagation of trees by axillary shoots

					Performance or comparison with other systems				
Species	Common name	Plant material	No. of clones	Type of CIS bioreactor	Proliferation	Rooting	Acclimation	Observations	Reference
*Castanea sativa* x *C. crenata*	Chestnut	Adult trees	8	10 L vessel	High	60–70% (ex vitro rooting plus acclimation)		Forced ventilation, rockwool as support. No comparison with SS	55
*Castanea spp*.	Chestnut	Adult trees	15	10/16 L vessel	High	70% (in vitro rooting plus acclimation)		Forced ventilation, CO_2_‐enriched air, rockwool, photoautotrophy. No comparison with SS	53
*Eucalyptus camaldulensis*	Eucalyptus	Adult trees	1	0.5 L flask	CIS > TIS > SS	100%	76%	Forced ventilation, hyperhydricity.	48
*E. camaldulensis*	Eucalyptus	n. s.	n. s.	0.37 L flask	n. s.	CIS > SS	90–100%	Natural ventilation, CO_2_‐enriched air, plastic or vermiculite support, photoautotrophy	49
*E. urophylla* x *E. grandis*	Eucalyptus	n. s.	n. s.	Miracle Pack and Vitron	High	High	100%	Natural ventilation, CO_2_‐enriched air, photoautotrophy	50
*Malus domestica*	Apple	CCP	1	5 L glass balloon with net	CIS > TIS	n. s.	n. s.	More hyperhydricity than in TIS. Physiological analysis. No comparison with SS	30, 71
*Macadamia tetraphylla*	Macadamia	Grafted seedlings	n. s.	Small flask	n. s.	100%	n. s.	Natural ventilation, vermiculite, CO_2_‐enriched air, photoautotrophy. No comparison with SS	52
*Samanea saman*	Rain tree	Seedlings	n. s.	0.24 L flask	High	High	n. s.	Natural ventilation, vermiculite, CO_2_‐enriched air, photoautotrophy. No comparison with SS	51

CCP, characterized commercial plants; CIS, continuous immersion; n.s., not specified; SS, semisolid medium; TIS, temporary immersion

Photoautotrophic micropropagation (PAM) was also investigated in CIS in other species as rain tree 51, macadamia 52, and chestnut 53. In the case of rain tree and macadamia, small flasks with natural ventilation were used (Figure 1C), whereas chestnut was cultured in large flasks with forced ventilation (Figure 1E). Chestnut is a tree important for its fruits and timber. As European chestnut (*C. sativa*) is currently being threatened by ink disease (caused by *Phytophthora cinnamomi* and *P. cambivora*), resistant hybrids of European and Asian chestnut (*Castanea sativa* x *C. crenata*, *C. sativa* x *C. mollissima*) should be propagated vegetatively. Chestnut is difficult to root, mainly when the plant material is of mature origin 54, and the aim of the application of CIS was to obtain high number of shoots of good quality to undergo the rooting process 53. In a first study, we used 10 L bioreactors adapted from food containers, with rockwool cubes for support, and applied forced ventilation in photomixotrophic conditions (adding sugar to the medium) 55. Then, we proliferated and rooted chestnut by PAM 53. After a short dip with auxin, the shoots were inserted in rockwool cubes containing medium without sugar. The cubes were placed in 16 L vessels with forced ventilation with CO_2_‐enriched air 53. This way, more than 6000 vigorous shoots belonging to 15 genotypes were evaluated, and rooting and acclimation rates of more than 70% were obtained, significantly improving the performance of this difficult‐to‐root species regarding conventional micropropagation.

As a resume, hyperhydricity (reported in one third of the studies) was the main obstacle for successful application of CIS methodology. However, many examples (∽56%) of applying CIS to culture of axillary shoots of trees described high proliferation and good percentages of rooting and acclimation. It is worth noting that in most of these successful cases shoots were cultured in PAM 49, 50, 51, 52, 53, emphasizing the advantage of obtaining a “natural” physiological state for a good transition to ex vitro conditions 43, 56.

### 3.2 Axillary shoots cultured by temporary immersion

Table 2 includes 25 published articles dealing with the utilization of TIS for axillary shoot‐based tree production. Eleven families and 16 genus are represented, including species growing in natural forests and in plantations. Most of these references use adult trees or rejuvenated material obtained from characterized commercial plants, whereas six studies refer exclusively to seedlings. The most popular devices listed in this table are vessels in which the liquid medium reaches the explants driven by filtered air entering the system, including bioreactors derived from the two‐flask system (40%), followed by commercial RITA^®^ (36%), and Plantform™ (16%) (Figures 2 and 3). Only two studies (8%) report the use of rocker bioreactors (Figure 4). Comparisons between TIS and SS performance are frequent, but less than 15% of reports analyzed more than one design of TIS 57, 58, 59. The use of TIS focused in the multiplication phase of micropropagation in 84% of references, and 88% of them reported the rooting of propagated plant material. Frequently (64%) the rooting phase occurred inside the bioreactors, whereas in other studies (24%) shoots produced by TIS were submitted either to in vitro rooting in SS or to ex vitro rooting. Acclimation success is mentioned in the 76% of the reports, as only six of them did not provide data on plant adaption to ex vitro conditions.

**Table 2:** Application of TIS to the propagation of trees by axillary shoots

					Performance or comparison with other systems				
Species	Common name	Plant material	No. of clones	Type of TIS bioreactor	Proliferation	Rooting^a^^,^ b	Acclimation	Observations	Reference
*Betula pendula, B. pubescens*	Birch	CCP	2	Two‐flasks	Species specific	TIS∼SS^a^	TIS∼SS	Slight hyperhydricity	70
*Castanea sativa* x *C. crenata* *C. sativa* x *C. mollissima*	Chestnut	Adult trees	10	RITA Plantform (PF)	PF > RITA > SS	PF > RITA > SS^b^	PF > RITA > SS	Hyperhydricity (controlled using rockwool as support)	58
*Cedrela odorata*	Spanish red cedar	Seedlings + adult trees	High number	BioMINT®	TIS > SS	TIS > SS^a^	98%	Juvenile > Mature material. No forced ventilation	42
*Corylus spp*. (Hybrids)	Hazelnut	CCP	4	Liquid Lab Rocker™	TIS > SS	TIS < SS^a^	n. s.	No forced ventilation	41
*Crescentia cujete*	Calabash tree	Seedlings	n. s.	RITA	TIS > CIS > SS	TIS > CIS, SS^a^	TIS (75%) > CIS, SS	Tree with medicinal properties	65
*Eucalyptus spp*. and hybrids	Eucalyptus	CCP	6	RITA	TIS > SS	TIS > SS^a^	TIS > SS	Hyperhydricity (controlled by manipulation of immersion). Genotypical differences	60
*E. camaldulensis*	Eucalyptus	Seedlings	n. s.	Two‐flasks (20 L) + additional aeration	TIS > CM	TIS > CM^a^	TIS > CM	Photoautotrophy; Florialite as support in TIS and CM	63
*E. camaldulensis*	Eucalyptus	Seedlings	n. s.	Two‐flasks (4 L) + additional aeration	TIS > SS	TIS > SS^a^	TIS > SS	Photoautotrophy; Vermiculite and paper pulp as support in TIS and agar in SS	61
*E. nitens*	Eucalyptus	Seedlings	n. s.	Two‐flasks	n.s.	TIS > SS^a^	>70%	TIS only during rooting	62
*Handroanthus heptaphyllus*	Black lapacho	Seedlings	n. s.	Two‐flasks	TIS > SS	TIS > SS^a^	TIS > SS	Tree with medicinal properties	68
*Ilex paraguariensis*	Yerba mate	CCP	n. s.	Two‐flasks	TIS > CIS, SS	TIS > SS^a^	80%	Tree with medicinal properties	66
*Malus domestica*	Apple M9	CCP	1	Ebb & flood	TIS ∼ SS	>90%^b^	>90%	Hyperhydricity controlled with aeration. Rooting by hydroponic culture	71
*M. domestica*	Apple M26	CCP	1	RITA	TIS > SS	>90%^a^	High	Hyperhydricity (controlled by manipulation of immersion)	72
*M. domestica*	Apple	CCP	1	PA‐TIS (two‐flasks)	n. s.	60%^a^	n. s.	Photoautotrophy	107
*Olea europaea*	Olive	CCP	n. s.	LifeReactor©, in‐house design	TIS ∼ SS	n. s.	n. s.	Hyperhydricity, sometimes contamination	59
*O. europaea*	Olive	CCP	n. s.	RITA	TIS > SS	n. s.	n. s.	Improvement of leaf characteristics	80
*O. europaea*	Olive	CCP	1	Plantform	TIS > SS	n. s.	n. s.	Cost reduction due to less requirement of zeatin	81
*Pistacea spp*.	Pistachio	Seedlings, adult trees	4	RITA	TIS > SS	50–70%^a^^,^ b	70–90%	Hyperhydricity (controlled by manipulation of immersion)	75
*Populus deltoides* x *P. trichocarpa*	Poplar	CCP	3	Two‐flasks	n. s.	TIS (97%) > SS a	TIS > SS	Photoautotrophy, mycorrhization	67
*Prunus avium* (and hybrid rootstocks)	Cherry	Adult trees	4	Two‐flasks	TIS > SS	TIS (100%) > SS^b^	TIS > SS	Hyperhydricity in some genotypes	76
*P. cerasifera*	Myrobolan	Young trees	1	RITA	TIS > SS	TIS > SS^a^	>80%	More [photosynthetic pigments] in RITA	69
*Quercus robur*	Oak	Seedlings	n. s.	Plantform	TIS ∼ SS	TIS∼SS^b^	n. s.	Hyperhydricity (controlled by manipulation of immersion)	77
*Salix viminalis*	Willow	Adult tree	1	RITA, Plantform (PF)	PF > RITA > SS	100%^a^	100%	Spontaneous rooting in all systems	57
*Tectona grandis*	Teak	Greenhouse tree	n. s.	Two‐flasks	TIS > SS	TIS (95 %) > SS^a^^,^ b	100%	Hyperhydricity (controlled by lowering cytokinin). Spontaneous rooting in TIS	73
*T. grandis*	Teak	Adult trees	n. s.	RITA	TIS > SS	TIS∼SS (∼90 %)^b^	TIS∼SS (∼90 %)	Hyperhydricity (controlled by lowering cytokinin, nº immersions, explant density)	74

a Rooting occurred in TIS.

b Shoots proliferated by TIS were rooted in SS or *ex vitro*.

CCP, characterized commercial plant; CIS, continuous immersion; CM, conventional micropropagation with supports different from agar; n. s., not specified; TIS, temporary immersion; SS, semisolid medium.

As with continuous immersion, eucalyptus is the most commonly studied plant and possibly the one in which the most advantageous results were obtained. As mentioned above, these trees are recalcitrant to vegetative propagation, mainly due to difficulties during rooting and acclimation. These problems have been addressed by the use of different TIS with forced ventilation, such as RITA^®^ 60 and various designs of the two‐flask system 61, 62, 63 (Figures 5 and 6). Bioreactors were used either for multiplication and rooting 60, 61, 63 or only for the phase of rooting 62. As reported by McAlister et al., great differences among clones regarding proliferation, rooting, and acclimation were detected 60. However, once hyperhydricity was controlled by optimizing immersion frequency, and duration 60, an overall increase in both proliferation and plant quality was obtained by the use of TIS 60, 61, 63.

The improvement of shoot quality observed by the use of RITA^®^ 60 was attributed to the air supply inside the bioreactors, which can reduce the internal humidity and favor gas exchange (O_2_, CO_2_, and ethylene) between the plant and the surrounding environment. The promotion of normal metabolism of plant tissues (aerobic respiration and photosynthesis), which allows the acquisition of a photoautotrophic state and later facilitates the transition to ex vitro conditions, is one of the claimed benefits of using ventilated vessels 43, 56, 64. Indeed, eucalyptus shoots cultured in a two‐flask system subjected to PAM (without sugar supplementation and with CO_2_ enriched air; Figure 6), showed higher photosynthetic rates and epicuticular leaf‐wax contents (as well as better stomatal function) than shoots cultured in conventional SS 61. Moreover, gas exchange did not only favor the development of the aerial part of the plant, as rooting percentages and/or root quality were also improved by the use of TIS during the rooting phase 60, 62, 63. The supply of O_2_ in the rooting zone promoted the development of normal roots directly from the base of the stems, without callus interference 60. The formation of well‐developed roots enhances the nutrient/water uptake rate of plants during the acclimation process, which was easier in eucalypt plants cultured by TIS than in those cultured in SS 60, 62, 63.

Besides eucalyptus, other tree species showed better rooting in bioreactors than in SS, as reported for calabash tree 65, yerba mate 66, poplar 67, black lapacho 68, and myrobolan 69. In the two later cases, the use of liquid medium significantly reduced the apical necrosis detected when shoots grew in agar‐based medium, thereby increasing the number of shoots that could be rooted. Shoots of easy‐to‐root trees, as birch 70, willow 57, and two apple rootstocks 71, 72 showed high frequencies of rooting in TIS (90–100%). Although in these cases the rooting results were similar to those obtained in SS, shoots rooted by TIS were easier to manage and more cost‐effective. Successful acclimation was reported in all these examples.

In other plants, shoots obtained by TIS were rooted ex vitro, as reported for chestnut 58 and teak 73, 74, or in vitro by using SS, as reported for pistachio 75, cherry 76, and oak 77. Except for oak shoots, the use of TIS increased the number and quality of shoots destined to rooting, improving the overall process with regard to conventional micropropagation. Pistachio, a tree important for its fruits, was cultured in RITA^®^ using juvenile and mature material of different species. As reported above for black lapacho 68 and myrobolan 69, pistachio shows apical necrosis when its shoots are proliferated in semi‐solid medium 78. TIS decreased the incidence of this disorder (thereby increasing the number of shoots suitable for rooting), once hyperhydricity was controlled by adjusting the duration and frequency of immersion 75.

In the case of chestnut, the aim of the application of TIS was to reduce micropropagation costs and too obtain high number of shoots of good quality for rooting 58. Since many chestnut genotypes are prone to developing hyperhydric symptoms even when cultured in semi‐solid medium 79, controlling this disorder was the major challenge faced in the application of TIS 58. In other species as eucalyptus, apple, teak, and pistachio 60, 72, 74, 75, hyperhydricity could be controlled by adjusting the duration and frequency of immersion. However, the only way to obtain a good proportion of normal chestnut shoots in bioreactors was to maintain the explants in an upright position during immersion, which was accomplished by using rockwool cubes as support material 58. This enabled successful propagation of ten ink‐resistant genotypes, which generally proliferated better in TIS than in semi‐solid medium. Plantform™ and RITA^®^ bioreactors were compared during the proliferation phase 58. Longer and more vigorous shoots were obtained in Plantform™ vessels, probably because these bioreactors are larger than RITA^®^, have larger headspace, and additional aeration can be supplied without immersion of the medium (Figures 2 and 3). Chestnut shoots cultured in Plantform™ were subjected to ex vitro rooting and the rooting + acclimation success (ranging from 40 to 80% depending on the genotype) was higher than obtained with SS.

Plantform™ bioreactors were also tested with other emblematic trees, such as olive and oak, although these protocols have still to be fully developed. Olive (*Olea europaea* L.) is mainly cultivated in the Mediterranean basin and is used both for oil extraction and table consumption. This tree is recalcitrant to micropropagation due to low (and cultivar‐dependent) proliferation rates, as well as low rooting and acclimation rates. The first attempts to culture olive in several TIS devices were hindered by low proliferation, contamination and high hyperhydricity 59. The use of RITA^®^ vessels enabled more shoots to be obtained than in semi‐solid cultures 80. Recently, healthy shoots of cv. Canino were produced in Plantform™ bioreactors using only half of the zeatin previously reported 81, thus potentially reducing production costs. Regarding pedunculated oak, a report of the proliferation of juvenile material of *Quercus robur* was recently published 77. Hyperhydricity was controlled by adjusting the immersion and aeration frequencies, and rooted shoots were obtained. Although the results were similar to those obtained in SS, the study findings demonstrated the feasibility of culturing axillary shoots of this tree in bioreactors.

TIS without ventilation (Figure 4) have also been applied to trees of economic importance for their fruits or wood, although with less frequency than the previous designs using forced ventilation. Hybrid hazelnut and Spanish red cedar were cultured in two different rocker system bioreactors named respectively Liquid Lab Rocker™ 41 and BioMINT^®^ 42. By this approach, as outlined before, the liquid medium does not reach the explants forced by air entering the flask but by mechanical movement of the bioreactor (Figure 4). Hazelnut and cedar shoots proliferated more by TIS than by SS, showing higher content of photosynthetic pigments 41, as well as larger leaves and more vigorous shoots 41, 42. Although hazelnut shoots rooted better when adventitious rooting was induced in SS than by TIS without ventilation 41, the rocker system did enhance root formation and acclimation (up to 98%) in the case of *Cedrela odorata* 42.

As a resume, most of the examples (∽80 %) of applying TIS to culture of axillary shoots of trees described higher proliferation by this method than by conventional micropropagation. High percentages of rooting and/or high quality of the rooted shoots (which in turn facilitated acclimation success) were reported in a similar range of studies (∽80%), although clear comparisons with SS were not provided in all of them. As previously observed in CIS, the main hindrance to overcome was hyperhydricity (reported in ∽45% of the studies), which was controlled mainly by lowering cytokinin supply and by manipulation of immersion. It is worth noting that significant reduction of production costs were reported 60, 63, 71, 74, 81.

### 3.3 Somatic embryos cultured by temporary and continuous immersion

Recent reviews dealing with design and use of bioreactors for embryo culture are available 27, 33, 34, but those focusing in tree culture are relatively scarce 35. Table 3 includes 22 published articles dealing with the utilization of TIS and CIS for somatic embryo‐based tree production, most of them regarding angiosperm cultures (∽75%). Twelve genus are represented, and only in four of them somatic embryos were derived from mature or characterized commercial plants, whereas in the rest the explants were obtained from embryonic or unspecified material. The most popular devices listed in this table are commercial RITA^®^ vessels (∽40%) and bioreactors derived from the two‐flask system (∽30%) (Figures 2 and 5). These apparatus are followed by airlift (Figure 1B) and stirred tank (Figure 1A) bioreactors (20 and 10%), together with other devices sometimes designed specifically for particular plants, as in the case of coffee 29, 82, 83, 84, 85, 86. Comparisons between bioreactors and SS are less frequent than in the case of axillary shoots, as are only shown in less than half of the references.

**Table 3:** Application of bioreactors to the propagation of trees by somatic embryos

					Performance or comparison with other systems^a^^,^ b					
Species	Common name	Plant material	No. of clones	Type of bioreactor	Proliferation	Maturation	Germination/Plant conversion	Acclimation	Observations	Reference
*Abies nordmanniana*	Nordmann fir	Embryonic	1	Two‐ flasks (TIS)	TIS > SS	TIS > SS	n. s.	n. s.	TIS promoted maturation	70
*Carica papaya*	Papaya	Embryonic	n. s.	RITA (TIS)	SS	SS	TIS (95 %) > SS	n. s.	TIS used for germination of mature embryos	89
*Castanea dentata* (and hybrids)	American chestnut	Embryonic	n. s.	Airlift (CIS)	TIS > SF	TIS > SF	n. s.	n. s.	Used for obtaining targets for genetic transformation	92
*Coffea arabica*	Coffee	Greenhouse plants	1	RITA (TIS)	TIS	TIS	75% ex vitro plant conversion plus acclimation		Physiological and chemical measurements	29
*C. arabica*	Coffee	Greenhouse plants	High number	RITA, MATIS, Two‐flasks (TIS)	SF	High	91%	High	Large‐scale propagation, histological and physiological measurements	85, 86
*C. canephora ∼ C.robusta*	Coffee	Greenhouse plants	17	Two‐flasks, box in bag (TIS)	SF	Two‐flasks (> 95 %)	46% ex vitro plant conversion plus acclimation		Large‐scale propagation, variability between batches	83
*C. canephora ∼ C.robusta*	Coffee	Greenhouse plants	n. s.	RITA, TRI‐bioreactor (TIS))	n.s.	TRI‐bioreactor (84 %) > RITA > SS		TRI‐bioreactor (89 %) > SS > RITA	Photoautotrophy, physiological measurements	82
*C. canephora ∼ C.robusta*	Coffee	Greenhouse plants	n. s.	Stirred tank (CIS), RITA, Two flask, box in bag (TIS)	Stirred tank	RITA, two flasks, box in bag		∽100%	Large‐scale propagation, photoautotrophy	84
*Hevea brasiliensis*	Rubber tree	Embryonic	n. s	∽RITA (TIS)	TIS > SS	TIS > SS	TIS^a^^ ^> SS	n. s.	TIS promoted synchronization of embryo development	87
*Kalopanax septemlobus*	Kalopanax	Grafted material	n. s.	TIS and CIS with net and forced ventilation	High	High	TIS > SS > CIS	100%	The use of a net improved TIS	88
*Picea abies*	Norway spruce	Embryonic	4	Two‐ flasks (TIS)	High	High	High/Medium	n. s.	Genotypical differences	101
*P. mariana, P. glauca‐engelmannii*	Black and interior spruce	Embryonic	2	Air‐lift, Stirred tank (CIS)	High	TIS > SS^b^	n. s.	n. s.	Maturation was higher when embryos were previously cultured in airlift bioreactors	102
*P. sitchensis*	Sitka spruce	Embryonic	2	Stirred tank, Air‐lift, Bubble, Hanging stirrer bar (CIS)	High	Better in bubble bioreactors	n. s.	n. s.	Interaction bioreactor type/embryogenic line	103
*Pinus kesiya*	Khasi pine	Embryonic	n. s.	Bubble bioreactor (CIS)	TIS > SF	TIS > SF^b^	TIS ∽SF	n. s		104
*Psidium guajava*	Guava	Embryonic	n. s.	RITA (TIS)	n. s.	TIS > SS^a^		n. s.		90
*Quercus robur*	Pedunculate oak	Mature trees	2	RITA (TIS)	TIS > SS	TIS < SS^a^	TIS > SS^b^	95%	High genotypical differences	108
*Q. robur*	Pedunculate oak	Seedlings, Mature trees	4	RITA (TIS)	TIS > SS	TIS > SS^b^	TIS > SS^a^^,^ b	TIS > SS	Selection phase of genetic transformation	93, 94
*Q. suber*	Cork oak	Embryonic	n. s.	RITA (TIS)	TIS∼SS	n. s.	n. s.	n. s.		109
*Santalum album*	Sandalwood	n. s.	n. s.	Airlift (CIS)	High	n. s.	n. s.	n. s.	Metabolite production	110
*Theobroma cacao*	Cacao	Mature trees	1	Two‐flasks (TIS)	TIS > SS	TIS > SS^a^		Good	Biochemical analysis, direct sowing of germinated embryos	91

a Process carried out in bioreactors

b Process carried out with material previously cultured in bioreactors

CCP, characterized commercial plant; CIS, continuous immersion; n. s., not specified; TIS, temporary immersion; SF, Shaken flask; SS, semisolid medium

In angiosperm somatic embryos, the use of bioreactors focused in the multiplication phase of micropropagation in ∽80% of references, and 88% of them reported the plant conversion of propagated plant material. In some cases (68%) the last events of embryo development (maturation, germination, and plant conversion) occurred inside the bioreactors, whereas in other studies ( ∽20%) embryos produced in liquid medium were submitted either to in vitro maturation in SS or to ex vitro germination and plant conversion. Acclimation success is mentioned in ∽60% of the reports. Maybe coffee is the plant that has benefitted more from the development of temporary immersion bioreactors for large‐scale propagation at industrial level 86. Somatic embryos of selected clones derived from the two main commercial species, *Coffea arabica* and *C. canephora* were successfully cultured by using a combination of bioreactors of different shape and volume. Embryos of *C. arabica* and/or *C. canephora* (Robusta) were cultivated by CIS in shaken flasks and in a stirred tank 83, 84, 85 and by TIS using 1 L RITA^®^ bioreactors, the 5 L MATIS^®^, two‐flask bioreactors, the TRI‐bioreactor (Figure 6) and the 10 L Box in Bag disposable bioreactor 29, 82, 83, 84, 85, 86. A high culture density positively affected embryo morphology by enhancing embryonic axis elongation, which allowed direct sowing of pre‐germinated embryos, greatly reducing the handling time. By the use of bioreactors handling decreased as plant production increased, allowing large‐scale propagation and successful industrial transfers to growers in Latin America, Africa, and Asia 86.

In other plants as rubber tree, kalopanax, papaya, guava, and cacao improvements in embryo germination and plant conversion were observed 87, 88, 89, 90, 91, although its commercial application did not reach the level of success of coffee.

Bioreactors were also used to improve genetic transformation protocols. The production of transgenic lines of several *Fagaceae* species, such as *Castanea dentata* 92 and *Quercus robur* 93, 94, was increased by culturing somatic embryos either in airlift bioreactors previously to transformation events 92, or by applying RITA^®^ to select kanamycin resistant transformants after *Agrobacterium* inoculation 93, 94. In the case of oak, transgenic embryos were obtained faster and in higher frequencies than in SS. Since phenolics and other growth inhibitors diffuse faster in liquid medium 95, those exuded by non‐resistant dying cells were probably rapidly diluted to innocuous levels, thereby minimizing negative effects on growth of transgenic cells. Other advantage of using RITA^®^ for oak transformation was that this bioreactor facilitated the plant conversion of transgenic lines originated from mature oak trees, both when the embryos were transferred to plates for maturation and germination treatments and when the embryos were maintained in the bioreactors 93.

In the case of conifers, however, the maturation, germination and plant conversion of embryos in liquid medium still remains as a challenge. Although there is a general agreement about the advantages of applying bioreactors to large‐scale gymnosperm production 96, 97, 98, currently the proliferation of cultures of these trees in liquid medium is mostly carried out using small flasks on rotary shakers 99, 100. Bioreactors have been used for the multiplication phase of somatic embryos of genus *Abies*, *Picea*, and *Pinus* 70, 101, 102, 103, 104, among other conifers. However, for maturation, germination and plant conversion, using either SS or different types of bioreactors are required 95, 96, 98, as current methods of proliferation in bioreactors can lead to problems such as failure to establish polarity or hyperhydricity 98. Also, light availability should be improved, as light is a critical factor especially during germination 98.

In general, the application of TIS to angiosperm embryo culture produced cases of clear success. Together with coffee, in which the use of various bioreactors allowed large‐scale propagation, Table 3 reports other examples in which TIS improved embryo quality. However, for the application of this technology to conifer production it is necessary to obtain synchronous cultures with well‐established polarity and competence to undergo plant conversion 98. This requires to solve some problems as the control the shear stress, which can damage the growing cells 27, 35, and to provide homogenous light to all the embryos 98.

## 4 ADVANTAGES, DISADVANTAGES, AND PROSPECTS OF THE USE OF BIOREACTORS

**Figure 7:**
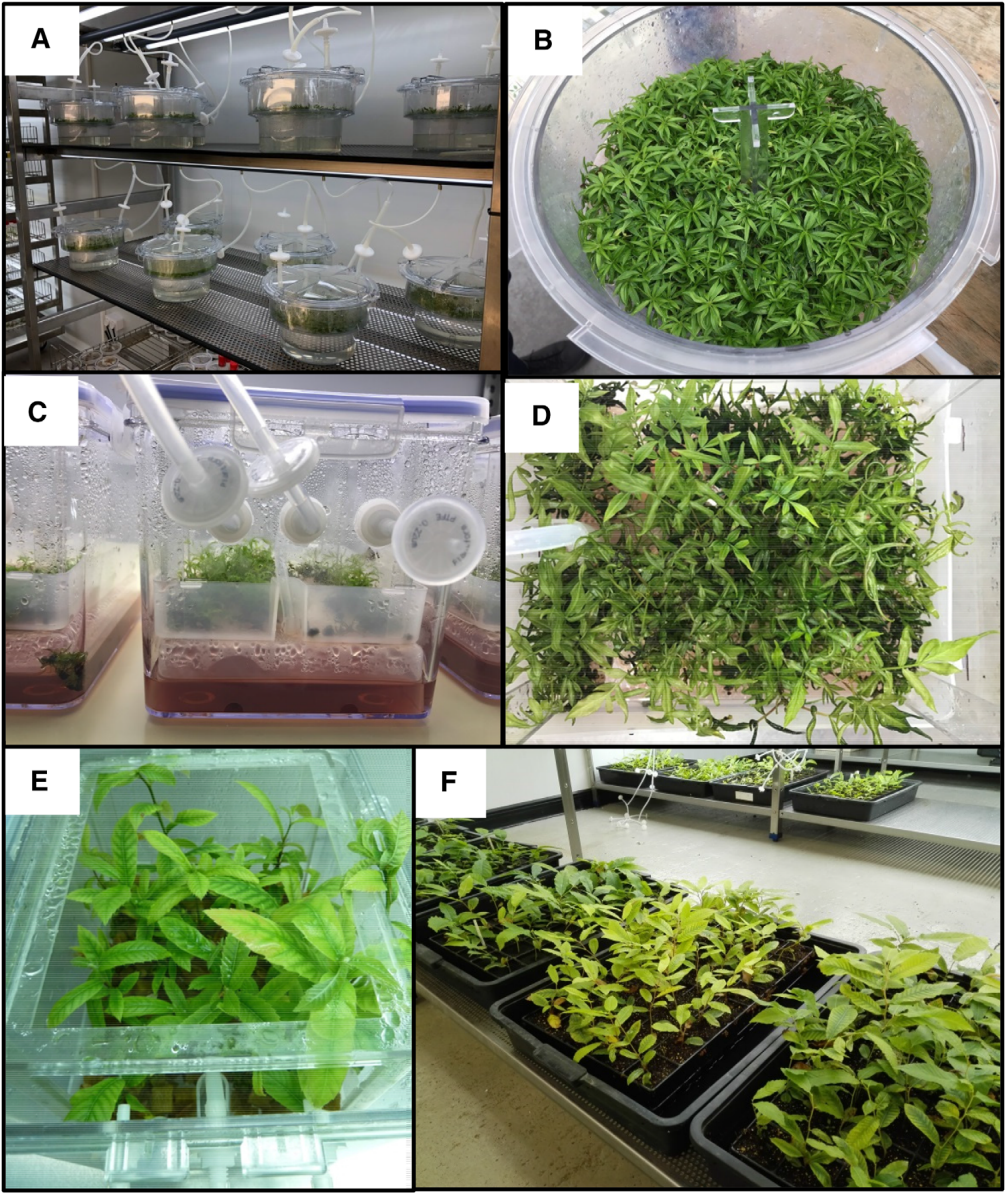
Industrial applications of bioreactors. (A, B) *Prunus* rootstocks cultured in MATIS^®^ bioreactors by Agromillora group. (C, D) Pistachio shoots cultured in Plantform™ by Vitrosur Lab SLU. (E, F) Chestnut shoots cultured by TRAGSA after being proliferated in Plantform™ (E) and during the acclimation phase (F)

For several years, the use of bioreactors has become part of the daily routine in most plant tissue culture laboratories. These systems can improve proliferation, rooting, plant conversion and acclimation of a wide range of plants, including trees The industrial applications of bioreactors are widespread, and besides the references in which participation of companies is cited 53, 55, 58, 60, 76, 83, 84, 85, 86, many companies culturing woody plants use bioreactors at experimental or commercial levels. Figure 7 shows some examples of the propagation of woody plants in bioreactors on an industrial scale.

However, it seems that the use of bioreactors to improve the propagation of forest trees has not yet reached its full potential. The decision as to whether to use bioreactors or not, and which type of bioreactor to use, is not always a matter of matching the characteristics of the bioreactor to the characteristics of the plant to be propagated, as economic concerns must also be taken into account. Although the use of bioreactors reduces the costs of consumables and personnel once the protocols are optimized 60, the initial outlay (for vessels, bombs, electrovalves, filters, etc.) is high. Commercial bioreactors are expensive and some parts have to be replaced after a few autoclaving cycles. Scientists working in research centres or in companies often have to make their own designs or improvise solutions using materials or devices designed for different purposes. The availability of cheap, customized bioreactors that could be acquired worldwide in a relatively short time would facilitate the implementation of liquid culture in many laboratories. Advances in 3‐D printing technology may allow the development of new and affordable designs on demand in the near future 105.

Bioreactors cannot be easily used with all types of plants. Problems such as hyperhydricity frequently arise during the development of new protocols in liquid medium for some species or genotypes, thus compromising the efficiency of the procedures 28, 58, 71, 106. Although physiological studies have been carried out in several species 29, 30, 61, 82, 85 there is necessary to get deeper knowledge of the physiological behavior of shoots and embryos in liquid medium.

In addition, although not always reported, the contamination risk has been highlighted in large‐scale somatic embryo and axillary shoot cultures 24, 28, 58, 59. Although contamination rates may be similar in bioreactors and in small vessels, bacteria and fungi proliferate faster in liquid medium. Greater losses occur due to contamination of larger vessels than with small flasks, which can discourage researchers from using bioreactors. Contamination depends not only on maintaining good laboratory practices during the work carried out in flow cabinets, but also on the environmental conditions and location of laboratory facilities (proximity to fields, ventilation, humidity, etc.). These conditions do not affect all bioreactor systems and all stages of experimental work to the same extent. Mireia Bordas (Agromillora Group) did not report any contamination problems when using MATIS® in experimental trials of culture of woody plants. Despite the rather excessive price of some components, the company plans to produce woody plants grown in bioreactors in the near future, especially for the pre‐acclimation phase (M. Bordas, pers. comm.). Beatriz Cuenca, a scientist working for TRAGSA nursery (Spain), has used Plantform™ bioreactors to multiply chestnut shoots 58 before rooting them photoautotrophically in a CIS 53 for more than 5 years. However, part of the multiplication phase has had to be performed in semi‐solid medium, after the Plantform™ bioreactors were severely affected by fungal contamination. This was probably influenced by the age of the facilities, which are in process of renewal, and their location, as they are surrounded by fields and forests (B. Cuenca, pers. comm.). Fortunately, the larger containers (without sugar) used for rooting were not as badly affected and were able to be used to improve chestnut acclimation. Susana Vilariño (Vitrosur Lab SLU) reported the use of “two‐flask system”, SETIS™ and Plantform™ to obtain eighty per cent of the annual production of eucalyptus and pistachio (S. Vilariño, pers. comm.). A common opinion among the three companies is that bioreactors can be used to complement semi‐solid culture, but not as a substitute. Aspects to consider before opting to use bioreactors include the excessive price of bioreactors and some components, such as spare parts and filters, and the need for careful training of operators in the correct installation and use of the devices.

## 5 CONCLUDING REMARKS

Bioreactors are useful tools for tree micropropagation and for the study of plant functioning. Use of these devices can help overcome the recalcitrance of some species and genotypes to proliferation, rooting, plant conversion, and acclimation. In addition, they can also be used to reduce the cost of large‐scale propagation. The number of tree species cultured in bioreactors is increasing steadily, and frequently the physiological state of plant propagules improves with these systems of culture, which also facilitate photoautotrophic propagation. However, two main types of challenges are still unresolved. At scientific level, it is necessary to unravel the physiological causes of hyperhydricity and to solve the difficulties to achieve maturation and plant conversion (especially in conifers). Besides, other issues such as the excessive cost and lack of availability of particular designs must be resolved to enable the general application of this promising technology.

## CONFLICT OF INTEREST

The authors have declared no conflict of interest.
